# Safety and Immunogenicity of Different Formulations of Norovirus Vaccine Candidate in Healthy Adults: A Randomized, Controlled, Double-Blind Clinical Trial

**DOI:** 10.1093/infdis/jix572

**Published:** 2017-11-13

**Authors:** Geert Leroux-Roels, Jakob P Cramer, Paul M Mendelman, James Sherwood, Ralf Clemens, Annelies Aerssens, Ilse De Coster, Astrid Borkowski, Frank Baehner, Pierre Van Damme

**Affiliations:** 1 Center for Vaccinology, Ghent University and University Hospital, Belgium; 2 Takeda Pharmaceuticals International AG, Zurich, Switzerland; 3 Takeda Vaccines Inc, Boston, Massachusetts; 4 Centre for the Evaluation of Vaccination, Vaccine and Infectious Disease Institute, University of Antwerp, Belgium

**Keywords:** norovirus, vaccine, immunogenicity, reactogenicity, adults

## Abstract

**Background:**

We investigated safety and immunogenicity of 1–2 doses of different bivalent virus-like particle (VLP) norovirus vaccine candidate (NoV) formulations in healthy 18- to 64-year-olds.

**Methods:**

On days 1 and 28, participants (n = 420) randomized to 14 equal groups received intramuscular control vaccine (hepatitis A) or 1 of 11 NoV formulations containing varying dosages of GI.1 and GII.4c genotype VLP antigens with aluminum hydroxide [Al(OH)_3_], and 0 μg, 15 μg, or 50 μg monophosphoryl lipid A (MPL). Immunogenicity was assessed on days 1, 28, 56, 208 and 393. Solicited local and systemic reactions were recorded for 7 days, unsolicited adverse events (AEs) until day 56, and serious AEs throughout the trial.

**Results:**

All NoV formulations induced similar increases in pan-immunoglobulin, immunoglobulin A, and histo-blood group binding antigen-blocking antibodies by day 56, mostly after 1 dose, that persisted above baseline to day 393. Higher GI.1 content interfered with GII.4c responses, and responses did not benefit from MPL. Overall reactogenicity consisted of mainly mild injection site pain, headache, and fatigue. No vaccine-related serious AEs were reported.

**Conclusions:**

All candidate NoV formulations were well tolerated. Overall, 15 μg GI.1/50 μg GII.4c elicited the best balance of immunogenicity with no clear benefit of MPL, and is the candidate formulation being taken forward in clinical development.

**Clinical Trials Registration:**

NCT02038907.

## Teaser

Bivalent (GI.1 and GII.4) virus-like particle norovirus candidate vaccine formulations were well tolerated and immunogenic, 1 dose inducing immune responses that persisted up to 1 year, which were not increased by inclusion of monophosphoryl lipid A or administration of a second dose.

Since their identification in the late 1960s, noroviruses (NoVs) have emerged as the single most significant cause of epidemic outbreaks of nonbacterial gastroenteritis worldwide [1], probably due to improvements in recognition rather than increased occurrence [2]. Noroviruses are responsible for a high burden of incapacitating illness affecting all ages, but with highest morbidity and potentially fatal consequences in the elderly, the very young, and those with chronic underlying medical conditions [3, 4]. To date, 7 genogroups and >40 genotypes based on the capsid have been identified, GI and GII genogroups being responsible for most disease [4]. Norwalk virus was the first identified norovirus, the GI.1 genotype of the GI genogroup [5]. However, since the mid-1990s, the GII.4 genogroup has been the principal cause of human disease [6] and, despite yearly drift of norovirus strains, GII.4 genotypes remain dominant and responsible for most outbreaks worldwide.

High transmissibility and difficulties in prevention of transmission by physical methods make vaccination the most promising measure to prevent NoV infection, especially in high-risk groups. Vaccine development has been hindered by the lack of suitable small animal models and difficulties encountered in propagating the virus in model systems, but virus-like particle (VLP) preparations have shown encouraging results in human challenge studies against both GI.1 [7] and GII.4 [8] genotypes.

Takeda’s intramuscular bivalent vaccine candidate is based on adjuvanted norovirus VLP antigens: the Norwalk GI.1 strain VLP that cross-reacts with other GI.1 strains [9], and a consensus strain (GII.4c) derived from 3 GII.4 strains—2006a (Yerseke), 2006b (Den Haag), and 2002 (Houston)—intended to provide broad coverage against GII strains [10].

Animal studies and human phase 1 and 2 trials have been conducted on the immunogenicity of candidate vaccine formulations adjuvanted with 50 µg monophosphoryl lipid A (MPL) and 500 µg aluminum hydroxide [10–13]. The primary goals of this trial were to evaluate the immunogenicity and safety of different candidate NoV vaccine formulations with varying dosages of GI.1 and GII.4c VLPs, aluminum hydroxide [Al(OH)_3_] and MPL adjuvants, and 1 dose vs 2 doses, in healthy adults aged 18–64 years.

## METHODS

### Study Design and Subjects

This phase 2, double-blind, controlled trial was performed in 2 centers in Belgium (Center for Vaccinology, Ghent University and the Center for the Evaluation of Vaccination, University of Antwerp) from 28 March 2014 to 19 June 2015. The protocol was approved by the ethics committee of each site and conducted in compliance with the International Council for Harmonisation Good Clinical Practices E6 guideline and applicable regulatory requirements. The trial objective was to select the optimal vaccine candidate formulation from different concentrations of VLP with Al(OH)_3_, with or without MPL, for further development, based on the safety, tolerability, and immunogenicity in adults.

Subjects were 420 healthy male and female volunteers, aged 18–64 years, enrolled in 2 equal age strata—18–49 and 50–64 years inclusive (n = 210 each). All participants provided written informed consent prior to initiation of any trial procedures and were required to be able to comply with all trial procedures and be available for the duration of the trial. Subjects were in good health at the time of study entry as determined by medical history, physical examination (including vital signs), and the clinical judgment of the investigator. Exclusion criteria included any serious chronic medical condition, known sensitivity to any of the vaccine components, or any recent or regular therapy likely to interfere with immune responses. Female volunteers were not breastfeeding, had a negative pregnancy test at screening, and practiced approved contraceptive methods until 6 months after the last vaccination.

NoV candidate formulations contained 15 μg, 50 μg, or 150 μg of each VLP with 0, 15 μg, or 50 μg of MPL and 167 μg or 500 μg Al(OH)_3_ per 0.5-mL dose. Participants were randomized to 14 equal groups (Table 1), respecting the 2 age strata, to receive either 1 dose or 2 doses, 28 days apart, of a respective formulation with hepatitis A vaccine (Havrix, GlaxoSmithKline, Wavre, Belgium) as control in single-dose groups to maintain the blinding (Table 1). A core factorial design of groups 1–7, 8A, and 9 included formulations with 3 VLP dosages in balanced and unbalanced combinations and the 3 MPL dosages in a 1-dose regimen.

**Table 1.** Compositions of the Different Norovirus Candidate Formulations and the Study Groups, With Attrition During the Trial

	Study Group													
Formulation	1	2	3	4	5	6	7	8		9	10		11	
								8A	8B		10A	10B	11A	11B
Norovirus vaccine composition, dosage in μg														
GI.1 VLP	15	15	50	15	15	50	15	15	15	50	50	50	15	15
GII.4c VLP	15	50	50	15	50	50	15	50	50	50	150	150	50	50
MPL	50	50	50	15	15	15	0	0	0	0	0	0	0	0
Al(OH)_3_	500	500	500	500	500	500	500	500	500	500	500	500	167	167
No. at day 1	30	30	30	31	30	31	30	32	28	29	30	29	29	31
Age 17–49 y	15	14	15	15	15	15	15	16	14	15	15	15	15	16
Age 50–64 y	15	16	15	16	15	16	15	16	14	14	15	14	14	15
Male:female	12:18	10:20	10:20	9:22	11:19	8:23	15:15	14:18	12:16	9:20	7:23	9:20	13:16	13:18
Day 1 vaccine^a^	Control	Control	Control	Control	Control	Control	Control	Control	Noro	Control	Control	Noro	Control	Noro
Day 28 vaccine^a^	Noro	Noro	Noro	Noro	Noro	Noro	Noro	Noro	Noro	Noro	Noro	Noro	Noro	Noro
No. at day 208	30	30	30	31	30	30; 1 subject withdrew	30	32	28	29	30	29	28; 1 subject withdrew	29; 1 AE, 1 loss to follow-up
No. at day 393	29; 1 subject withdrew	29; 1 subject withdrew	30	31	30	29; 1 subject withdrew	29; 1 adverse event	32	28	29	30	29	28	29

^a^Control = Havrix.

Abbreviations: AE, adverse event; Al(OH)_3_, aluminum hydroxide; MPL, monophosphoryl lipid A; Noro, norovirus virus-like particle formulation; VLP, virus-like particle.

### Immunogenicity

Sera were prepared at days 1, 28, 56, 208, and 393 to assess humoral immunity. Enzyme-linked immunosorbent assay (ELISA) pan-immunoglobulin (Ig) and immunoglobulin A (IgA) antibodies were measured as described previously [11]; in addition, histo-blood group binding antigen (HBGA) blocking titers (BT_50_), which have been suggested to provide a serologic correlate of protection following NoV vaccination [14], were measured at each time-point [15].

### Safety and Tolerability

Safety was assessed using subject-completed diary card to solicit local reactions and systemic adverse events (AEs) for 7 days postvaccination. Solicited local reactions—pain, erythema, swelling, and induration—were assessed by severity: pain, according to the degree of interference with daily activity, others as measured diameters with a supplied ruler. Solicited systemic AEs included fever (oral temperature), headache, fatigue, myalgia, arthralgia, vomiting, and diarrhea, which were also classified by severity. Unsolicited AEs were recorded on the diary cards from day 1 to day 28; serious adverse events (SAEs) and AEs of special interest (eg, immune-mediated AEs) were reported throughout the trial.

### Statistical Analysis

The primary immunogenicity endpoints were seroresponse rate (SRR) and the percentage of subjects with a ≥4-fold rise in pan-Ig titers against GI.1 and GII.4c at day 56. SRR was also assessed at days 208 and 393. IgA and by HBGA antibody titers for GI.1 and GII.4c were also assessed and summarized and analyzed overall and by age cohort. Primary analysis was performed in the per protocol set, defined as all subjects who were randomized and received both doses of vaccine and had evaluable blood samples at baseline and within the specified day 56 window, with no major protocol violations.

Descriptive summaries were generated for each individual study group. For the core factorial design of groups 1–7, 8A, and 9, summaries and analyses were generated for overall groupings by: MPL dosage aggregated over VLP formulation; VLP antigen formulation (15/15, 15/50, or 50/50) aggregated over MPL dosage; GI.1 VLP dosage (15 μg or 50 μg) aggregated over MPL and GII.4c VLP dosages; and GII.4 VLP dosage (15 μg or 50 μg) aggregated over MPL and GI.1 VLP dosage. For these groupings, formal statistical inference was performed using Fisher exact test for SRR endpoints and analysis of covariance (ANCOVA) models for log-transformed titers, at a .05 level of significance without multiplicity adjustment. The ANCOVA models included the age stratum and MPL or VLP dosage as factors as well as the log-transformed baseline titer as a covariate.

## RESULTS

Of 451 volunteers screened and randomized, 420 enrolled and received their first injections on day 1; 412 (98.1%) completed all planned trial visits up to day 393 (Table 1). Eight subjects did not complete the trial; 5 withdrew themselves, 1 was lost to follow-up, and 2 had AEs considered unrelated to the study procedures that ultimately resulted in the deaths of the 2 participants (see “Safety and Tolerability” below).

### Immunogenicity

Unsurprisingly, in view of the small number of dropouts, per protocol analyses were not substantially different from the full analysis set (data not shown).

### Pan-Ig Responses

Table 2 shows responses in each of the 14 study groups against both vaccine genotypes as pan-Ig geometric mean titers (GMTs). All groups displayed marked increases in antibodies against both VLP antigens 4 weeks after a single NoV dose. Baseline GI.1 pan-Ig levels were similar across study groups, as were GII.4c baseline levels, which tended to be higher than those against GI.1; this was not unexpected given that the main circulating strains currently are GII.4 strains [16]. Postvaccination levels increased to similar extents by day 56 after all groups had received at least 1 dose of NoV. Titers waned by days 208 and 393, but remained higher than baseline levels in all groups. GMTs were 3- to 4-fold higher against GI.1 than GII.4c, and this difference was maintained through day 393. In the 2-dose groups (8B, 10B, 11B), the second NoV dose given 4 weeks after the first dose did not increase either the magnitude or the persistence of the response in healthy adults.

**Table 2.** Pan-Immunoglobulin Geometric Mean Titers Against GI.1 and GII.4c at the Indicated Time-points in Each Study Group in the Overall Cohort (Per Protocol)

Time-point	Study Group													
	1	2	3	4	5	6	7	8		9	10		11	
								8A	8B		10A	10B	11A	11B
ELISA pan-immunoglobulin (G1.1)														
Day 1	833 (470–1479)	752 (424–1333)	678 (377–1219)	612 (324–1156)	625 (417–938)	520 (288–938)	891 (498–1594)	843 (474–1497)	742 (389–1414)	707 (423–1181)	827 (460–1488)	898 (544–1482)	991 (471–2082)	727 (444–1191)
Day 28	799 (450–1420)	695 (397–1219)	574 (318–1036)	763 (397–1465)	574 (377–873)	515 (281–941)	910 (525–1579)	822 (465–1451)	14489 (9465–22178)	666 (388–1144)	781 (425–1436)	20839 (16138–26910)	1038 (503–2142)	15973 (11478– 22229)
Day 56	14418 (9900–20998)	14171 (9631–20853)	17505 (12021–25490)	13686 (8666–21612)	13982 (10197–19172)	18266 (14181–23528)	16083 (12246–21122)	15974 (9733–26217)	**12613 (8897–17881**)	25809 (17950–37108)	15466 (10836–22075)	**17696 (13819–22661**)	13389 (8411–21313)	**12236 (9263–16163**)
Day 208	5725 (4151–7896)	5070 (3587–7165)	6278 (4414–8931)	5499 (3766–8029)	4468 (3418–5839)	5106 (3902–6683)	5661 (4203–7623)	7175 (5179–9940)	6863 (4994–9433)	7537 (5444–10433)	5361 (3887–7395)	7459 (6067–9171)	5166 (3656–7299)	5725 (4452–7362)
Day 393	3886 (2566–5884)	2975 (2039–4340)	4774 (3335–6835)	4896 (3284–7299)	3210 (2266–4547)	3875 (2715–5528)	4940 (3497–6978)	4773 (3373–6755)	4716 (3300–6740)	5478 (3704–8103)	3543 (2502–5016)	5687 (4235–7635)	3990 (2627–6060)	4256 (3148–5756)
ELISA pan-immunoglobulin (GII.4c)														
Day 1	1107 (622–1970)	1203 (750–1929)	1445 (829–2519)	995 (586–1690)	922 (601–1414)	1131 (669–1912)	1340 (793–2265)	1239 (757–2026)	978 (587–1629)	1280 (788–2077)	1473 (888–2444)	1324 (837–2096)	1983 (1354–2905)	1618 (1019–2570)
Day 28	1017 (536–1930)	1171 (733–1869)	1493 (872–2557)	987 (601–1620)	839 (554–1271)	1098 (652–1848)	1268 (780–2063)	1311 (796–2158)	8459 (6242–11465)	1219 (753–1974)	1495 (986–2266)	12902 (9700–17161)	1934 (1309–2858)	10877 (7643–15478)
Day 56	4596 (3049–6928)	8637 (6576–11344)	5750 (4273–77397)	4046 (2823–5799)	7545 (5276–10791)	7803 (6017–10119)	3445 (2309–5138)	9946 (6706–14750)	**6622 (4862–9020**)	5130 (3392–7759)	10624 (7979–14146)	**9996 (7499–13322**)	9328 (7094–12266)	**8164 (5964–11176**)
Day 208	2341 (1405–3902)	3653 (2690–4961)	2807 (1691–4658)	2630 (1807–3826)	2985 (2084–4274)	3212 (2303–4480)	2251 (1467–3455)	5018 (3454–7288)	3838 (2780–5299)	2550 (1679–3874)	4531 (3368–6097)	4694 (3302–6673)	4413 (3136–6209)	4350 (3195–5922)
Day 393	2237 (1341–3730)	2962 (1934–4537)	2384 (1565–3634)	1973 (1340–2903)	1946 (1381–2743)	2174 (1484–3184)	2267 (1442–3566)	2970 (2018–4371)	2425 (1710–3439)	1871 (1153–3035)	2550 (1873–3472)	3133 (2210–4442)	2706 (1844–3972)	3007 (2119–4266)

Values in parentheses are 95% confidence intervals. Values in shaded boxes are 4 weeks after administration of a norovirus vaccine formulation; bolded values are 4 weeks after a second dose of norovirus vaccine formulation.

Abbreviation: ELISA, enzyme-linked immunosorbent assay.

**Figure 1.**
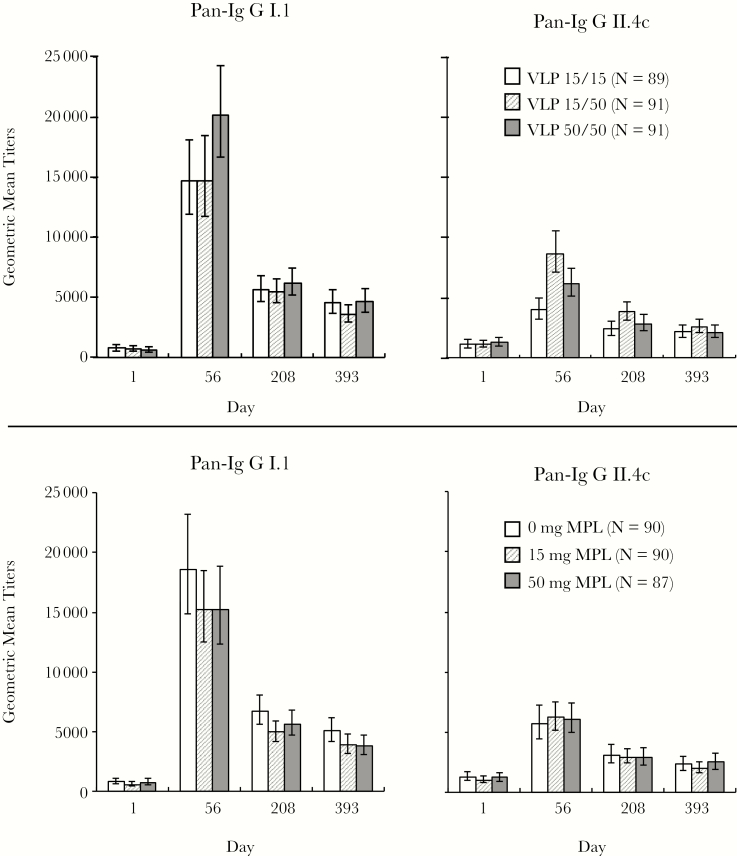
Geometric mean titers (95% confidence interval) of pan-immunoglobulin (Pan-Ig) against GI.1 and GII.4c virus-like particle (VLP) antigens. Upper panels show grouping by VLP antigen dosages, irrespective of the inclusion or dosage of monophosphoryl lipid A (MPL) (VLP 15/15 = groups 1, 4, and 7; VLP 15/50 = groups 2, 5, and 8A; VLP 50/50 = groups 3, 6, and 9). Lower panels show grouping by MPL dosage irrespective of VPL composition (0 μg MPL = groups 7, 8A, and 9; 15 μg MPL = groups 4, 5, and 6; 50 μg MPL = groups 1, 2, and 3).

Pan-Ig responses were similar irrespective of formulation, with group GMTs ranging from 13686 to 25809 ELISA units (EL.U)/mL for GI.1, and 3445 to 12902 EL.U/mL for GII.4c after 1 dose. Levels then waned to similar extents across groups. Data were combined for factorial analysis of study groups with the same formulations with respect to VLP contents (Figure 1). The combined data illustrate that varying GI.1 VLP from 15 μg to 50 μg had little effect on GI.1 GMTs. However, increasing the GII.4c VLP content from 15 μg to 50 μg, although not affecting the GI.1 response, did result in a significant increase in anti-GII.4c pan-Ig, a difference that persisted until day 393. Furthermore, increasing the GI.1 content from 15 μg to 50 μg appeared to counteract this increase in the GII.4c response.

A similar factorial analysis for the 0-, 15-, or 50-μg dosages of MPL showed no demonstrable increase in response due to MPL (Figure 1). Rather, GI.1 responses were lower in the presence of MPL, while GII.4c responses were similar with or without MPL. Nor did inclusion of MPL affect the persistence of pan-Ig antibodies at day 393.

A second vaccination (groups 8B, 10B, and 11B) did not further increase pan-Ig responses, GMTs after the second dose being lower than after the first (Table 2). Similarly, no differences in responses were seen between groups given formulations containing 167 μg (groups 11A and 11B) rather than 500 μg of Al(OH)_3_ (groups 8A and 8B) without MPL, their GMTs being similar to other groups.

Seroresponse rates ranged from 80.0% to 100% for GI.1, and from 33.3% to 83.3% for GII.4c across study groups (Table 3). Combining groups according to VLP dosage showed no differences in GI.1 SRR between those who received 15 μg (52.2%) or 50 μg (52.9%) of GI.1 VLP. However, the SRR with 50 μg GII.4c VLP (60.7%) was significantly higher than with 15 μg (36.0%, *P* < .001). Differences in GI.1 SRR between groups combined for 0 μg, 15 μg, or 50 μg of MPL were not significant. A small but statistically significant (*P* = .049) increase in GII.4c SRR was observed between the 0-μg (50.0%) and 15-μg (65.6%) groups, but 50 μg of MPL did not significantly increase the SRR compared with either the 0-μg or 15-μg groups.

**Table 3.** Seroresponse Rates for Pan-Immunoglobulin Against GI.1 and GII.4c at Days 28 and 56 (Per Protocol)

Antigen	Day	Study Group													
		1	2	3	4	5	6	7	8		9	10		11	
									8A	8B		10A	10B	11A	11B
Pan-immunoglobulin seroresponse rates per study group															
GI.1	28	0	0	0	6.9 (0.8–22.8)	0	0	0	0	85.7 (67.3–96.0)	0	0	93.1 (77.2–99.2)	4.2 (0.1–21.1)	93.3 (77.9–99.2)
	56	80.0 (61.4–92.3)	89.7 (72.6–97.8)	100 (87.7–100)	89.7 (72.6–97.8)	93.3 (77.9–99.2)	96.8 (83.3–99.9)	86.7 (69.3–96.2)	87.5 (71.0–96.5)	85.7 (67.3–96.0)	92.9 (76.5–99.1)	90.0 (73.5–97.9)	96.6 (82.2–99.9)	83.3 (62.6–95.3)	86.7 (69.3–96.2)
GII.4c	28	0	0	0	0	0	0	3.3 (.1–17.2)	3.1 (.1–16.2)	78.6 (59.0–91.7)	0	3.3 (.1–17.2)	86.2 (68.3–96.1)	0	70.0 (50.6–85.3)
	56	50.0 (31.3–68.7)	65.5 (45.7–82.1)	46.4 (27.5–66.1)	41.4 (23.5–61.1)	83.3 (65.3–94.4)	71.0 (52.0–85.8)	33.3 (17.3–52.8)	71.9 (53.3–86.3)	78.6 (59.0–91.7)	42.9 (24.5–62.8)	73.3 (54.1–87.7)	72.4 (52.8–87.3)	54.2 (32.8–74.49)	63.3 (43.9–80.1)
Seroresponse rates by VLP dosage															
GI.1	56	15 μg GI.1 VLP = 52.2 (44.7–59.7)					50 μg GI.1 VLP = 52.9 (41.9–63.7)					15 μg vs 50 μg, *P* > .999			
GII.4c		15 μg GII.4c VLP = 36.0 (26.0–46.8)					50 μg GII.4c VLP = 60.7 (53.1–67.9)					15 μg vs 50 μg, *P* < .001			
Seroresponse rates by MPL dosage															
GI.1	56	0 μg MPL 88.9 (80.5–94.5)		15 μg MPL 93.3 (86.1–97.5)				50 μg MPL 89.7 (81.3–95.2)			0 μg vs 15 μg, *P* = .433; 0 μg vs 50 μg, *P* > .999; 15 μg vs 50 μg, *P* = .428				
GII.4c		0 μg MPL 50.0 (39.3–60.7)		15 μg MPL 65.6 (54.8–75.3)				50 μg MPL 54.0 (43.0–64.8)			0 μg vs 15 μg, *P* = .049; 0 μg vs 50 μg, *P* = .653; 15 μg vs 50 μg, *P* = .128				

Data are shown as percentage (95% confidence interval).

Abbreviations: MPL, monophosphoryl lipid A; VLP, virus-like particle.

### IgA Responses

Similar IgA response profiles were observed against GI.1 and GII.4c 4 weeks after vaccination, but responses were lower in magnitude than those measured as pan-Ig (Supplementary Table 1). IgA responses did not increase with the VLP dosage or following a second dose, when they were about half those observed after the first dose, nor were they affected by the presence or absence of MPL. IgA levels showed the same waning of titers after day 56 observed with pan-Ig titers, but levels persisted above baseline at day 393.

### HBGA-Blocking Antibodies

**Figure 2.**
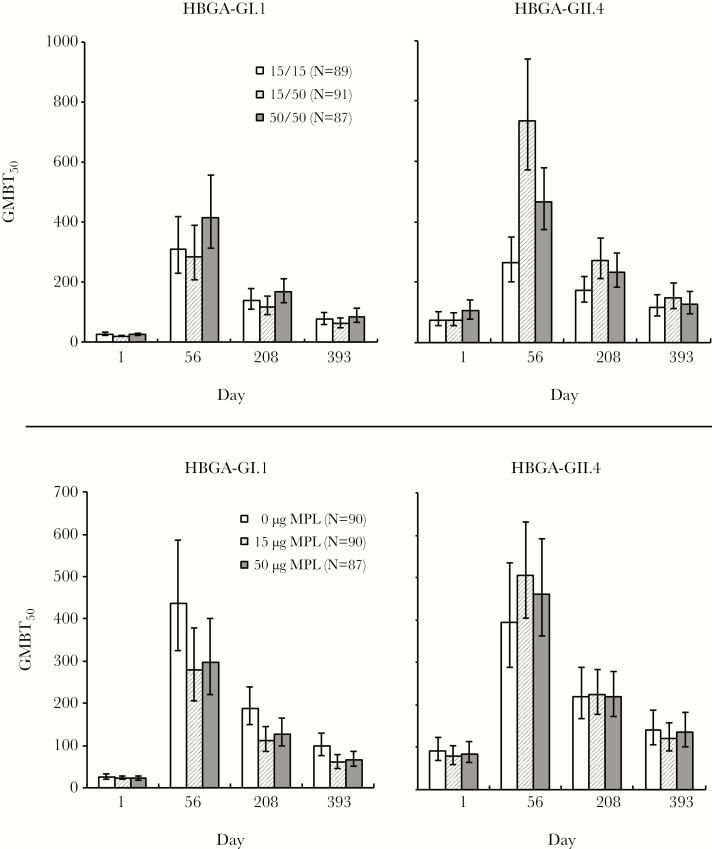
Geometric mean (95% confidence interval) of histo-blood group binding antigen (HBGA) antibodies (GMBT_50_) against GI.1 and GII.4c virus-like particle (VLP) antigens. Upper panels show grouping by VLP antigen dosages, irrespective of the inclusion or dosage of monophosphoryl lipid A (MPL) (VLP 15/15 = groups 1, 4, and 7; VLP 15/50 = groups 2, 5, and 8A; VLP 50/50 = groups 3, 6, and 9). Lower panels show grouping by MPL dosage irrespective of VPL composition (0 μg MPL = groups 7, 8A, and 9; 15 μg MPL = groups 4, 5, and 6; 50 μg MPL = groups 1, 2, and 3).

As with pan-Ig and IgA, there were no major differences in HBGA-blocking titers between individual groups (Table 4). HBGA-blocking titers also waned, but remained higher than baseline values in all groups at day 393. When combined for those groups who received the same GI.1/GII.4c VLP formulations or MPL dosages, HBGA-blocking immune profiles reflect the pan-Ig responses (Figure 2). HBGA-blocking antibodies against GI.1 were slightly higher when 50 μg of GI.1 was used, an effect that persisted to day 393. However, increasing the dosage of GII.4c from 15 μg to 50 μg had a more marked effect on HBGA-blocking antibodies against GII.4c; an effect was partially blocked by the higher GI.1 dosage. Combining groups for MPL dosage showed both that MPL dosages decreased titers of HBGA-blocking antibodies against GI.1. A small increase in GII.4c responses with 15 μg MPL at day 56 was less with the higher dosage of 50 μg, and any increase did not persist to day 208 when neither the presence nor the dosage of MPL had any influence on the titers.

**Table 4.** Geometric Mean HBGA (BT_50_) Against GI.1 and GII.4c in Each Study Group in the Overall Cohort (Per Protocol)

	Study Group													
Time-point	1	2	3	4	5	6	7	8		9	10		11	
								8A	8B		10A	10B	11A	11B
HBGA-blocking antibody BT_50_ (GI.1)														
Day 1	24.8 (17.0–36.2)	17.9 (14.4–22.3)	27.1 (18.2– 40.4)	26.5 (16.6–42.4)	19.1 (15.8–23.2)	24.8 (18.3–33.8)	30.5 (20.1– 46.2)	24.5 (17.4–34.5)	23.1 (16.9–31.7)	24.2 (17.0–34.4)	23.2 (17.3–31.1)	22.3 (16.3–30.4)	29.6 (19.0–46.0)	19.8 (15.3–25.7)
Day 28	25.0 (17.5–35.7)	17.6 (14.3–21.9)	24.6 (17.1–35.3)	25.8 (16.4–40.6)	18.6 (15.2–22.7)	23.0 (17.2–30.9)	27.3 (19.5– 38.1)	25.1 (18.0–35.0)	348 (192–629)	26.0 (18.0–37.6)	24.8 (18.0–34.3)	386 (236–631)	30.9 (20.2–47.3)	382 (236–617)
Day 56	281 (168–468)	236 (132–423)	403 (242–673)	264 (1429–492)	229 (137–381)	356 (220–578)	400 (255–627)	413 (235–726)	**399 (271–585**)	510 (288–903)	247 (156–392)	**464 (337–638**)	286 (150–544)	**356 (250–507**)
Day 208	130 (82.4–205)	98.2 (61.2–158)	161 (101–258)	128 (77.8–212)	80.1 (50.2–128)	139 (96.4–201)	160 (110–232)	202 (131–312)	196 (131–295)	208 (130–332)	115 (77.0–170)	219 (160–301)	109 (64.9–184)	174 (125–243)
Day 393	67.8 (40.9–112)	46.9 (30.8–71.6)	88.9 (53.59– 148)	69.5 (39.8–121)	44.0 (27.9–69.4)	71.9 (45.1–115)	91.5 (59.2–141)	108 (65.4–177)	103 (60.7–176)	97.9 (56.2–171)	55.0 (35.6–85.1)	114 (72.1–179)	69.6 (38.6–126)	76.5 (51.5–114)
HBGA-blocking antibody BT_50_ (GII.4c)														
Day 1	66.0 (36.6–119)	76.9 (48.0–123)	119 (68.3–206)	78.6 (47.3–131)	60.9 (36.9–101)	96.6 (58.0–161)	80.7 (46.1–141)	89.8 (52.6–153)	56.1 (34.6–91.0)	103 (61.0–173)	102 (64.8–161)	92.0 (54.5–155)	118 (72.9–191)	126 (76.8–207)
Day 28	69.3 (39.3–122)	72.4 (44.4–118)	131 (76.6–225)	78.6 (48.2–111)	67.7 (41.3–111)	94.5 (55.3–161)	74.6 (43.8–127)	98.1 (54.5–177)	553 (334–913)	99.4 (58.8–168)	123 (79.4–192)	688 (445–1066)	112 (67.8–186)	883 (610–1278)
Day 56	337 (213–532)	686 (428–1102)	429 (305–602)	294 (197–439)	676 (437–1044)	632 (481–831)	189 (107–335)	843 (552–1287)	**427 (263–691**)	360 (213–607)	863 (564–1320)	**596 (400–889**)	710 (481–1048)	**604 (406–901**)
Day 208	187 (121–288)	242 (154–378)	237 (158–356)	187 (128–273)	230 (142–371)	260 (180–374)	143 (84–241)	347 (236–510)	278 (194–398)	205 (125–335)	341 (238–487)	351 (253–487)	289 (179–465)	329 (224–481)
Day 393	121 (69.7–208)	171 (101–292)	116 (68.0–198)	120 (72.9–198)	105 (62.3–176)	134 (81.3–222)	109 (62.0–193)	183 (115–290)	126 (77.1–206)	131 (78.8–219)	180 (127–254)	171 (105–279)	185 (110–311)	193 (118–314)

Values in parentheses are 95% confidence intervals. Values in shaded boxes are 4 weeks after administration of a norovirus vaccine formulation; values in bold are 4 weeks after a second dose of norovirus vaccine formulation.

Abbreviations: HBGA, histo-blood group binding antigen blocking antibodies.

**Figure 3.**
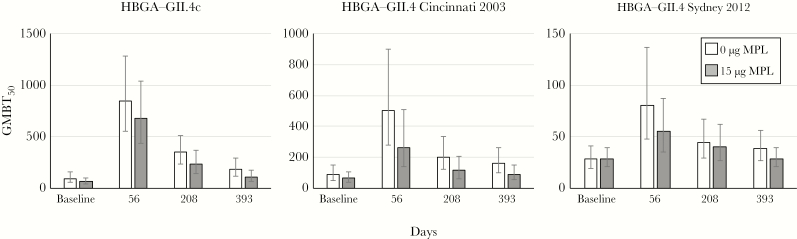
Geometric mean (95% confidence interval) of histo-blood group binding antigen (HBGA) antibodies (GMBT_50_) against different GII.4 antigens: the GII.4c virus-like particle (VLP) antigen and those from GII.4 Cincinnati 2003 and GII.4 Sydney 2012 strains. Sera tested were from group 5 (n = 30) and group 8A (n = 32), which had received 15 μg monophosphoryl lipid A (MPL) and no MPL, respectively, in formulations with 15 μg GI.1 and 50 μg GII.4c VLP antigen dosages.

The potential of MPL to improve cross-reactivity of the responses against nonvaccine antigens was investigated with recently circulating nonvaccine GII.4 strains, namely Cincinnati 2003 [8] and Sydney 2012 (Figure 3). Responses measured against these nonconsensus strains were lower than against GII.4c, and neither was increased by MPL in this study population; rather, there was a trend for higher responses against both GII.4 strains without MPL.

### Age-Related Differences in Responses

Although groups were smaller when the 2 age cohorts—18–49 and 50–64 years—were considered separately, the individual groups presented the same profile of responses as the total cohort (Supplementary Tables 2–5). Pan-Ig responses against GI.1 were similar in magnitude in both age groups, and higher in both age groups when no MPL was present. Anti-GII.4c responses were slightly lower in the older age group: without MPL, 6269 (95% confidence interval [CI], 4331–9073) in 18- to 49-year-olds vs 5132 (95% CI, 3719–7081) in 50- to 64-year-olds; with 15 μg MPL, 8726 (95% CI, 6752–11276) vs 4534 (95% CI, 3494–5883), respectively; and with 50 μg MPL, 7394 (95% CI, 5749–9510) vs 5090 (95% CI, 3802–6815). The presence of MPL had no statistically significant effect on GII.4c titers in either age group.

### Safety and Tolerability

Two subjects were withdrawn from the study due to AEs. Both participants eventually died of further complications of their medical conditions, although neither fatality was considered by the investigators to be associated with the study interventions. Further details are provided in the Supplementary Materials.

A further 21 subjects distributed across the study groups reported 23 SAEs up to day 393, none of which were considered to be related to the study procedures. The good tolerability of the candidate vaccine formulations is illustrated by the low rates of solicited local reactions and systemic AEs, comparable with the licensed and generally well-tolerated control vaccine Havrix (Supplementary Table 6). Local reactions consisted almost exclusively of injection site pain, reported after 44% of Havrix injections and 36%–48.5% of NoV candidate injections. The rate was slightly lower with the 0-μg MPL groups (38/91 [41.8%]) than the 15-μg MPL (50/92 [54.3%]) or 50-μg (49/90 [54.4%]) groups. One Havrix and 2 NoV injections caused pain described as severe. There were low rates of the other solicited local reactions, all but 1 with NoV candidates, which were not associated with any particular dosage of VLP or MPL.

Systemic AEs were reported at similar rates with either Havrix or first or second NoV doses (Supplementary Table 6). The most frequent were headache (16.3%–22.7%), fatigue (11.4%–21.6%), diarrhea (5.7%–11.4%), and myalgia (4.5%–11.4%). The majority were mild or moderate, and resolved within 2–3 days of the injection. Three reports of fever, 2 following Havrix and 1 after NoV, involved transient oral temperatures between 38°C and 39°C. There were 157 unsolicited AEs reported by 105 of 332 (31.6%) subjects vaccinated with Havrix, and 188 unsolicited AEs reported by 119 of 332 (35.8%) after NoV as second injection. Most of these were described as mild to moderate, with 3 (0.9%) and 11 (2.7%) severe AEs being reported after Havrix and NoV, respectively. In the 88 subjects who received 2 doses of NoV, 28 (31.8%) reported a total of 50 unsolicited AEs after the first dose, and 31 (35.2%) reported 42 unsolicited AEs after the second dose. These AEs were also mainly described as mild to moderate, with 2 and 3 reported as severe after the first and second NoV injections, respectively. Most of these unsolicited AEs were considered by the investigators to be unrelated to either Havrix (56%) or NoV (64%) administrations.

## DISCUSSION

This study was designed to establish the best NoV formulation with respect to VLP content, VLP ratio, MPL, and Al(OH)_3_ adjuvant content as well as dosing in 2 age cohorts. We also investigated the impact of a second dose of vaccine candidate, using a licensed hepatitis A vaccine to maintain the blinding. All tested NoV formulations were well tolerated with reactogenicity profiles similar to the hepatitis A vaccine. No safety signals were detected, irrespective of the presence or dosage of MPL adjuvant or the Al(OH)_3_ content. No vaccine-related SAEs or withdrawals due to vaccine-related AEs were reported.

All formulations elicited similar responses against GI.1 and GII.4c antigens measured as pan-Ig, IgA, and HBGA-blocking antibodies across study groups, irrespective of VLP or MPL contents or number of doses. Titers persisted above baseline values up to 1 year after administration. Assessment of individual study groups, or combined on the basis of their MPL content, found no evidence that MPL increased the response—rather, the trend was for lower pan-Ig and HBGA-blocking antibody responses to GI.1 in the presence of MPL. Furthermore, addition of MPL did not appear to improve the cross-reactivity assessed as the HBGA-blocking antibody responses to 2 different circulating GII.4 strains, although titers in the HBGA blocking assay are difficult to compare across genotypes. Work is ongoing to quantify vaccine HBGA responses against currently circulating virus strains. A similar analysis of the different respective VLP dosages found that increasing the GI.1 and GII.4c dosages produced incremental increases in both respective responses, but the higher dosage of GI.1 had a refractory effect on the GII.4c response, which is currently the predominant strain causing human illness. This suggests that the best balance of immune responses is achieved with a formulation containing 15 μg GI.1 VLP and 50 μg GII.4c VLP, confirming a previously reported study in 18- to 49-year-old adults using MPL-based formulations [13].

A specific combination of Al(OH)_3_ and MPL forms the adjuvant system 04 (AS04), used in GlaxoSmithKline Biologicals’ commercial VLP vaccine against human papillomavirus [17] and in 1 of its hepatitis B vaccines [18]. The NoV candidates all use Al(OH)_3_ to stabilize the VLPs and, in the absence of MPL, a reduction in the content did not affect the response. The addition of 15 μg or 50 μg MPL to the Al(OH)_3_-based formulations did not provide any improvements in terms of magnitude or persistence of antibody levels, or cross-reactivity to related but distinct norovirus strains. Indeed, there was a trend to lower responses to GI.1 in the presence of MPL. The lack of any apparent effect of MPL might be assumed to be due to differences in the formulation methodologies used to prepare ASO4 and the NoV formulations. However, the mouse potency assay that is used as a release test for all the NoV lots used in this study confirmed enhancement by MPL (data not shown). Therefore, the lack of effect in the presented nonnaive human study population did not appear to be a formulation issue.

One limitation of our study is that we did not assess any potential impact of MPL on the kinetics of immune responses, which were measured 4 weeks after vaccination. In a similar study population, MPL-containing formulations elicited robust responses after 7–10 days that persisted with slight waning to 28 days [13]. Nor have we attempted to select participants based on their baseline serostatus or to assess the impact of priming, with immunity presumably due to prior episodes of natural exposure to norovirus. Overall results were consistent when the results were looked at in terms of the 2 age cohorts in each group (18–49 years and 50–64 years), although the older cohort did display GII.4c responses that were lower in magnitude than in the younger group. These data show no major benefits of MPL or a second dose.

This study identified the formulation containing 15 μg GI.1 VLP and 50 μg GII.4c VLP with 500 mg Al(OH)_3_, but without MPL, as the most promising candidate to be taken further in development in healthy adults. This VLP composition is in agreement with results of previous studies that only looked at MPL-containing formulations [12, 13]. Antibody titers achieved with these formulations are consistent with those described previously with other candidate formulations. HBGA-blocking antibodies have been suggested to correlate with protection against norovirus illness [14], and titers ≥500 have been associated with protection against moderate-to-severe vomiting or diarrheal illness due to GII.4 genotype in a human challenge study [15]. While not directly comparable with the other study, due to differences in assay methods and laboratories, the HBGA GMTs achieved with the various formulations without MPL are of the same order of magnitude so may indicate that protection will be afforded. This will only be confirmed by larger full-scale efficacy trials of the selected candidate.

Although generally self-limiting in healthy adults, norovirus illness can be serious and cause complications and even death in older adults and those with chronic medical conditions [3, 4]. It is encouraging to note that the younger and older age cohorts in this study displayed similar immune responses to the different formulations, although further studies will be required in elderly cohorts who will also present the opposing complications of immunosenescence and immune memory of related strains. The current study allows development to focus on one candidate formulation in adults containing 15 μg GI.1 VLP and 50 μg GII.4c VLP and 500 μg Al(OH)_3_ in such future studies in healthy adults.

## Supplementary Data

Supplementary materials are available at *The Journal of Infectious Diseases* online. Consisting of data provided by the authors to benefit the reader, the posted materials are not copyedited and are the sole responsibility of the authors, so questions or comments should be addressed to the corresponding author.

Supplementary Material

## Notes

***Acknowledgments.*** The authors are grateful to all the volunteers who participated in the study, and to the staff at the 2 study centers. We also acknowledge the expert assistance of Fien De Boever and Leen Suykens for study coordination and the study investigator, Cathy Maes, MD. Keith Veitch of Takeda Vaccines assisted in preparation of the manuscript and provided general editorial assistance.

***Financial support.*** This work was supported by the study sponsor, Takeda Vaccines, Inc.

***Potential conflicts of interest.*** J. P. C., P. M. M., J. S., and A. B. are all full-time employees of the study sponsor, R. C. and F. B. were employees of the study sponsor at the time of the study. G. L.-R., A. A., I. d. C., and P. v.-D. have received grant support from Takeda Vaccines through their universities for the conduct of this study. R. C. is currently a consultant with GRID RIO Consulting, Rio de Janeiro, Brazil. F.B. is Area Head Prophylactic Vaccines, Clinical Development, CureVac AG, Frankfurt, Germany. All authors have submitted the ICMJE Form for Disclosure of Potential Conflicts of Interest. Conflicts that the editors consider relevant to the content of the manuscript have been disclosed.

Presented in part: IDWeek 2016, New Orleans, Louisiana, 26–30 October 2016. Abstract 1342.
