# Supplementary material for: Safety and Immunogenicity of Different Formulations of Norovirus Vaccine Candidate in Healthy Adults: A Randomized, Controlled, Double-Blind Clinical Trial
Source: J Infect Dis. 2017 Nov 13;217(4):597–607. doi: 10.1093/infdis/jix572 (PMC5853300; doi:10.1093/infdis/jix572)
Supplement: Supplementary Material [file jix572_suppl_supplementary_material.docx]

**Leroux-Roels et al.**

**Supplementary Materials**

**Description of fatalities**

**Tables 1 to 5** show Geometric Mean Titers (GMT) of the indicated antibodies.

Shaded values are those determined 4 weeks after administration of a NoV vaccine formulation

Shaded and outlined are those determined 4 weeks after administration of a second dose of NoV

nd = not determined.

* n = N-1 (no data for one sample)

** n = N-2 (no data for two samples)

**Supplemental Table 1.** IgA GMTs (95% CI) against GI.1 and GII.4c

**Supplemental Table 2.** Pan-Ig GI.1 GMTs (95% CI) at each time-point in the 18-49 and 50-64 year-old cohorts

**Supplemental Table 3.** Pan-Ig GII.4c GMTs (95% CI) at each time-point in the 18-49 and 50-64 year-old cohorts

**Supplemental Table 4.** Geometric HBGA BT_50_ (95% CI) against GI.1 at each time-point in the 18-49 and 50-64 year-old cohorts

**Supplemental Table 5.** Geometric HBGA BT_50_ (95% CI) against GII.4c at each time-point in the 18-49 and 50-64 year-old cohorts

**Supplemental Table 6.** Rates of solicited local reactions and systemic adverse events within 7 days of injection.

**Brief descriptions of two fatalities during the study**

Two fatalities occurred during the trial, neither of which was considered to be associated with study procedures or vaccine.

The first case was a 61-year-old man in Group 11B with a history of hepatitis C infection given two doses of NoV candidate (15μg GI.1, 50μg GII.4, 167μg Al(OH)_3_, no MPL) on March 31 and April 28, 2014, respectively. He was hospitalized twice for epigastric pain, in May and August 2014, and diagnosed with cholecystolithiasis in September 2014. He progressively deteriorated and died in November 2014 due to multiple organ failure associated with acute pancreatitis, consistent with his history of cholecystolithiasis.

The second case was a 62-year-old woman enrolled in Group 7 who received Havrix^®^ on April 10, 2014 and NoV candidate (15μg GI.1, 15μg GII.4, 500μg Al(OH)_3_, no MPL) on May 8, 2014. Following a diagnosis of cervical carcinoma in August 2014 she underwent repeated hospitalizations for pneumonia/COPD, urinary tract infection and a compression fracture, ultimately dying of cardiac arrhythmia in April 2015.

| **Supplemental Table 1. IgA GMTs** (95% CI) **against GI.1 and GII.4c** at the indicated days in each study group in the overall cohort (Per Protocol) | | | | | | | | | | | | | | |
| --- | --- | --- | --- | --- | --- | --- | --- | --- | --- | --- | --- | --- | --- | --- |
| **Group** | **1** | **2** | **3** | **4** | **5** | **6** | **7** | **8** | | **9** | **10** | | **11** | |
|  |  |  |  |  |  |  |  | **8A** | **8B** |  | **10A** | **10B** | **11A** | **11B** |
| **ELISA IgA – G1.1** | | | | | | | | | | | | | | |
| **N =** | 30 | 29 | 28 | 29 | 30 | 31 | 30 | 32 | 28 | 28 | 30 | 29 | 24 | 30 |
| **Day 1** | 52.9  (29.3–95.4) | 35.6  (22.0–57.6) | 45.2  (27.8–73.5) | 29.7  (15.7–56.4) | 30.1  (19.4–46.8) | 44.5  (23.0–86.2) | 53.6  (27.9–103) | 60.1  (30.7–118) | 73.3  (38.5–139) | 39.8  (21.1–75.2) | 58.5  (34.6–98.9) | 40.8  (23.5–70.7) | 54.4  (26.9–139) | 40.2  (24.5–66.1) |
| **Day 28** | 48.9  (26.8–89.0) | 32.5  (20.6–51.1) | 44.4*  (26.4–74.9) | 38.3  (21.7–67.7) | 29.4  (18.8–46.0) | 46.2  (23.6–90.3) | 54.4  (28.5–104) | 59.8  (30.9–116) | 1650  (892–3051) | 41.1*  (21.7–78.0) | 56.3  (33.7–94.1) | 2148  (1418–3254) | 63.5  (30.7–131) | 1703  (1182–2454) |
| **Day 56** | 1358  (808–2284) | 996  (539–1840) | 2223  (1468–3366) | 1016  (528–1954) | 1369  (816–2294) | 1850  (1188–2881) | 1595  (883–2881) | 1436  (711–2898) | 811  (416–1579) | 2074  (1168–3684) | 1511  (865–2637) | 923  (559–1522) | 1129  (598–2131) | 848  (524–1373) |
| **Day 208** | nd | nd | nd | nd | 303  (179–512) | nd | nd | 512  (271–969) | nd | nd | nd | nd | nd | nd |
| **Day 393** | nd | nd | nd | nd | 224  (154–326) | nd | nd | 340*  (206–561) | nd | nd | nd | nd | nd | nd |
| **ELISA IgA – GII.4c** | | | | | | | | | | | | | | |
| **N =** | 30 | 29 | 28 | 29 | 30 | 31 | 30 | 32 | 28 | 28 | 30 | 29 | 24 | 30 |
| **Day 1** | 83.4  (41.2–169) | 110  (61.9–194) | 163  (93.32–286) | 103  (60.6–174) | 72.8  (41.1–129) | 115  (65.7–200) | 100  (57.3–175) | 133  (75.7–234) | 106  (61.4–183) | 129  (74.3–224) | 159  (100–250) | 122  (70.9–210) | 200  (126–316) | 177  (103–303) |
| **Day 28** | 77.8  (38.7–156) | 106  (60.4–185) | 170*  (96.7–300) | 111  (64.4–191) | 73.8  (42.3–129) | 116  (67.1–202) | 106  (63.0–179) | 139  (75.4–257) | 954  (637–1427) | 120*  (68.0–212) | 165  (107–253) | 948  (667–1348) | 201  (128–316) | 1122  (748–1682) |
| **Day 56** | 496  (317–775) | 841  (563–1255) | 584  (420–812) | 433  (294–637) | 803  (518–1246) | 714  (515–990) | 337  (206–551) | 1089  (678–1749) | 579  (373–898) | 514  (306–864) | 1118  (739–1691) | 552  (384–794) | 973  (677–1398) | 691  (452–1057) |
| **Day 208** | nd | nd | nd | nd | 252  (151–420) | nd | nd | 428  (261–702) | nd | nd | nd | nd | nd | nd |
| **Day 393** | nd | nd | nd | nd | 140  (82.7–236) | nd | nd | 273*  (171–437) | nd | nd | nd | nd | nd | nd |

| **Supplemental Table 2. Pan-Ig GI.1 GMTs** (95% CI) at the indicated days in each study group in the 18-49 and 50-64 year-old cohorts (Per Protocol set) | | | | | | | | | | | | | | |
| --- | --- | --- | --- | --- | --- | --- | --- | --- | --- | --- | --- | --- | --- | --- |
| **Group** | **1** | **2** | **3** | **4** | **5** | **6** | **7** | **8A** | **8B** | **9** | **10A** | **10B** | **11A** | **11B** |
| **Pan-Ig vs GI.1 18-49 year-olds** | | | | | | | | | | | | | | |
| **N =** | 15 | 13 | 14 | 14 | 15 | 15 | 15 | 16 | 14 | 15 | 15 | 15 | 12 | 15 |
| **Day 1** | 507  (248–1036) | 768  (288–2048) | 579  (203–1646) | 500  (176–1424) | 548  (268–1122) | 332  (142–777) | 703  (330–1498) | 498  (177–1395) | 447  (188–1061) | 491  (214–1129) | 847  (397–1807) | 678  (413–1115) | 839  (219–3218) | 840  (375–1881) |
| **Day 28** | 490  (239–1003) | 696  (264–1831) | 443  (164–1197) | 696  (251–1932) | 525  (250–1102) | 320  (133–771) | 786  (390–1584) | 506  (179–1428) | 14018  (7371–26657) | 461  (199–1069) | 810  (384–1707) | 21034  (15294–28928) | 818  (248–2701) | 19143  (11952–30660) |
| **Day 56** | 17119  (239–1003) | 16291  (264–1831) | 14137  (164–1197) | 16396  (251–1932) | 14797  (250–1102) | 18431  (133–771) | 19314  (390–1584) | 11663  (179–1428) | 12706  (7371–26657) | 31032  (199–1069) | 16499  (384–1707) | 16201  (15294–28928) | 13926  (248–2701) | 13578  (11952–30660) |
| **Day 208** | 6285  (248–1036) | 6025  (288–2048) | 5760  (203–1646) | 4747  (176–1424) | 4984  (268–1122) | 4694*  (142–777) | 5906  (330–1498) | 7163  (177–1395) | 7029  (188–1061) | 7994  (214–1129) | 4970  (397–1807) | 6909  (413–1115) | 4429  (219–3218) | 6262*  (375–1881) |
| **Day 393** | 4128  (248–1036) | 3958*  (288–2048) | 4431  (203–1646) | 4670  (176–1424) | 3410  (268–1122) | 3309**  (142–777) | 4819  (330–1498) | 4710  (177–1395) | 4766  (188–1061) | 5131  (214–1129) | 3819  (397–1807) | 4941  (413–1115) | 3973  (219–3218) | 4525*  (375–1881) |
| **Pan-Ig vs GI.1 50-64 year-olds** | | | | | | | | | | | | | | |
| **N =** | 15 | 16 | 14 | 15 | 15 | 16 | 15 | 16 | 14 | 13 | 15 | 14 | 12 | 15 |
| **Day 1** | 1371  (553–3398) | 739  (340–1606) | 795  (400–1581) | 739  (309–1764) | 713  (444–1145) | 793  (337–777) | 1128  (330–1865) | 1428  (859–2373) | 1231  (456–3322) | 1074  (593–1947) | 808  (298–2192) | 1212  (470–3123) | 1169  (484–2828) | 629  (322–1228) |
| **Day 28** | 1303  (522–3250) | 695  (326–1481) | 759*  (366–1575) | 831  (320–2158) | 627  (380–1033) | 804  (339–1906) | 1054  (390–1584) | 1334  (816–2180) | 14975  (7828–28647) | 1057*  (549–2035) | 753  (259–2183) | 20632  (13113–32463) | 1317  (483–3593) | 13328  (8050–22067) |
| **Day 56** | 12143  (7235–20379) | 12654  (7509–21323) | 21675  (13503–34795) | 11562  (5816–22983) | 13213  (8033–21731) | 18113  (13613–24101) | 13392  (10319–17379) | 21879  (14942–32036) | 12521  (7348–21336) | 20865  (11034–39455) | 14499  (8563–24547) | 19453  (12325–30702) | 12872  (7043–23524) | 11027  (7258–16751) |
| **Day 208** | 5216  (3564–7633) | 4406  (2746–7069) | 6844  (3730–12558) | 6308  (3706–10737) | 4005  (2626–6109) | 5497  (3903–7743) | 5425  (3421–8603) | 7187  (4874–10595) | 6703  (3993–11251) | 7042  (4394–11285) | 5783  (3157–10594) | 8098  (5532–11854) | 6025  (4048–8968) | 5234*  (3400–8057) |
| **Day 393** | 3642*  (2022–6561) | 2401  (1368–4214) | 5144  (2964–8929) | 5117  (3074–8516) | 3021  (1767–5164) | 4405  (2920–6644) | 5074*  (2955–8711) | 4837  (3343–6999) | 4666  (2866–7597) | 5908  (3601–9694) | 3287  (1817–5944) | 6612  (4075–10727) | 4006  (2221–7226) | 4004*  (2367–6770) |

| **Supplemental Table 3. Pan-Ig GII.4c** **GMTs** (95% CI) at the indicated days in each study group in the 18-49 and 50-64 year-old cohorts (Per Protocol set) | | | | | | | | | | | | | | |
| --- | --- | --- | --- | --- | --- | --- | --- | --- | --- | --- | --- | --- | --- | --- |
| **Group** | **1** | **2** | **3** | **4** | **5** | **6** | **7** | **8A** | **8B** | **9** | **10A** | **10B** | **11A** | **11B** |
| **Pan-Ig vs GII.4c 18-49 year-olds** | | | | | | | | | | | | | | |
| **N =** | 15 | 13 | 14 | 14 | 15 | 15 | 15 | 16 | 14 | 15 | 15 | 15 | 12 | 15 |
| **Day 1** | 1129  (478–2666) | 1520  (772–2995) | 2056  (1072–3940) | 1227  (590–2550) | 1680  (992–2843) | 1365  (657–2837) | 1667  (867–3207) | 1097  (530–2270) | 1010  (415–2461) | 1351  (582–3135) | 2410  (1538–3774) | 1583  (869–2884) | 2039  (1167–3564) | 2458  (1242–4866) |
| **Day 28** | 1046  (435–2516) | 1443  (741–2808) | 2045  (1144–3657) | 1221  (597–2496) | 1522  (911–2541) | 1293  (626–2670) | 1648  (973–2792) | 1126  (591–2145) | 10560  (6567–16982) | 1320  (600–2904) | 2299  (1560–3389) | 15275  (10955–21300) | 1836  (1062–3173) | 17022  (12399–23368) |
| **Day 56** | 5559  3195–9669) | 9127  (5631–14793) | 8257  (6223–10955) | 5441  (3617–8186) | 12540  (7494–20982) | 9435  (6414–13877) | 4139  (2364–7246) | 8562  (3986–18392) | 8366  (5363–13051) | 6809  (3495–13266) | 15045  (10758–21039) | 11207  (8602–14601) | 8889  (5654–13973) | 12143  (8831–16698) |
| **Day 208** | 2869  (1495–5506) | 4413  (2913–6684) | 4507  (3195–6354) | 2529  (1559–4103) | 4479  (2592–7739) | 3339*  (1925–5793) | 2678  (1633–4392) | 4174  (2217–7858) | 4769  (2912–7812) | 2692  (1308–5540) | 5220  (3940–6914) | 5298  (3460–8111) | 3704  (2061–6657) | 5501*  (3561–8498) |
| **Day 393** | 2523  (1246–5110) | 3270*  (1582–6759) | 3558  (2428–5212) | 1986  (1143–3452) | 2826  (1733–4610) | 2179**  (1199–3962) | 2460  (1373–4406) | 2573  (1287–5141) | 2892  (1728–4842) | 1676  (782–3592) | 3354  (2518–4468) | 3493  (2432–5016) | 2264  (1152–4450) | 4304*  (2921–6342) |
| **Pan-Ig vs GII.4c 50-64 year-olds** | | | | | | | | | | | | | | |
| **N =** | 15 | 16 | 14 | 15 | 15 | 16 | 15 | 16 | 14 | 13 | 15 | 14 | 12 | 15 |
| **Day 1** | 1085  (450–2618) | 995  (488–2026) | 1016  (392–2636) | 819  (354–1895) | 506  (283–892) | 948  (415–2165) | 1078  (442–2629) | 1398  (663–2947) | 946  (499–1793) | 1202  (699–2068) | 901  (367–2212) | 1094  (504–2374) | 1929  (1045–3564) | 1066  (569–1996) |
| **Day 28** | 989  (347–2820) | 988  (485–2013) | 1064*  (397–2854) | 809  (378–1731) | 463  (270–793) | 942  (414–2143) | 976  (409–2326) | 1526  (664–3507) | 6777  (4517–10167) | 1104*  (602–2025) | 972  (474–1993) | 10766  (6519–17781) | 2037  (1065–3895) | 6951  (3897–12397) |
| **Day 56** | 3800  (1962–7360) | 8258  (5791–11775) | 4005  (2473–6486) | 3067  (1692–5566) | 4540  (3088–6675) | 6530  (4512–9451) | 2867  (1531–5366) | 11554  (8383–15923) | 5242  (3341–8223) | 3700  (2271–6030) | 7503  (4901–11486) | 8842  (5034–15530) | 9789  (6661–14387) | 5489  (3348–8998) |
| **Day 208** | 1910  (810–4506) | 3133  (1960–5008) | 1748  (676–4521) | 2727  (1454–5113) | 1989  (1301–3040) | 3105  (142–777) | 1892  (330–1498) | 6032  (177–1395) | 3088  (188–1061) | 2396  (214–1129) | 3934  (397–1807) | 4123  (413–1115) | 5257  (219–3218) | 3440*  (375–1881) |
| **Day 393** | 1965  (849–4549) | 2751  (1533–4937) | 1599  (758–3370) | 1960  (1065–3607) | 1341  (852–2110) | 2169  (1241–3790) | 2078  (949–4550) | 3429  2236–5257) | 2034  (1205–3432) | 2124  (1078–4184) | 1939  (1118–3861) | 2789  (1434–5421) | 3235  (2039–5130) | 2100  (1191–3702) |
| **Supplemental Table 4. Geometric HBGA BT_50_ (95% CI) against GI.1** at the indicated days in each study group in the 18-49 and 50-64 year-old cohorts | | | | | | | | | | | | | | |
| **Group** | **1** | **2** | **3** | **4** | **5** | **6** | **7** | **8A** | **8B** | **9** | **10A** | **10B** | **11A** | **11B** |
| **HBGA BT_50_ vs. GI.1 in 18-49 year-olds** | | | | | | | | | | | | | | |
| **N =** | 15 | 13 | 14 | 14 | 15 | 15 | 15 | 16 | 14 | 15 | 15 | 15 | 12 | 15 |
| **Day 1** | 18.9  (12.7–28.0) | 16.7  (13.3–21.0) | 23.3  (13.8–39.5) | 27.6  (11.8–64.8) | 18.4  (13.5–25.1) | 20.9  (14.1–31.1) | 26.9  (14.7–49.0) | 19.1  (13.2–27.6) | 21.1  (12.7–35.2) | 19.5  (12.8–29.8) | 19.7  13.2–29.3) | 16.5  (13.5–20.2) | 21.2  (14.0–32.1) | 20.2  12.7–32.2) |
| **Day 28** | 18.5  (13.0–26.3) | 16.4  (13.6–19.7) | 22.0  (14.0–34.5) | 25.9  (11.5–58.6) | 20.5  (14.0–29.9) | 21.0  (14.0–31.6) | 23.7  (15.2–37.1) | 20.5  (13.8–30.6) | 353  (160–775) | 21.3  (13.4–33.9) | 21.8  (13.9–34.3) | 339  (175–653) | 23.4  (15.2–36.2) | 322  (155–668) |
| **Day 56** | 284  (128–631) | 252  (90.8–697) | 287  (145–569) | 284  (109–737) | 244  (122–488) | 232  (112–479) | 461  (235–903) | 269  (108–672) | 434  (251–749) | 357  (168–758) | 232  (105–509) | 354  (224–557) | 291  (99.0–856) | 308  (183–517) |
| **Day 208** | 136  (67.1–275) | 101  (45.0–228) | 128  (65.1–253) | 131  (58.8–293) | 84.2  (42.0–169) | 109*  (63.7–187) | 159  (97.8–260) | 182  (89.3–370) | 228  (128–405) | 167  (89.1–311) | 114  (63.6–204) | 163  (106–251) | 101  (41.2–246) | 152*  (104–222) |
| **Day 393** | 62.4  (29.5–132) | 45.9*  (25.0–84.1) | 78.3  (40.7–150) | 71.4  (27.8–183) | 45.7  (23.7–88.4) | 45.0**  (21.4–94.9) | 79.9  (44.0–145) | 80.2  (33.2–194) | 127  (54.9–296) | 68.2  (30.5–153) | 55.1  29.2–104) | 76.1  (38.5–150) | 60.6  (24.4–151) | 69.7*  (39.2–124) |
| **HBGA BT_50_ vs. GI.1 in 50-64 year-olds** | | | | | | | | | | | | | | |
| **N =** | 15 | 16 | 14 | 15 | 15 | 16 | 15 | 16 | 14 | 13 | 15 | 14 | 12 | 15 |
| **Day 1** | 32.5  (16.6–63.5) | 19.0  (13.1–27.8) | 31.5  (16.2–61.1) | 25.5  (14.6–44.5) | 19.9  (15.1–26.1) | 29.2  (17.7–48.0) | 34.6  (18.2–65.7) | 31.5  (17.4–57.1) | 25.3  (16.3–39.1) | 30.9  (16.5–57.9) | 27.3  (17.2–43.4) | 30.8  (16.9–55.8) | 41.3  18.4–92.9) | 19.4  (14.4–26.1) |
| **Day 28** | 33.8  (18.0–63.4) | 18.8  (12.9–27.4) | 27.8*  (14.7–52.6) | 25.7  (14.8–44.5) | 16.9  (14.2–20.1) | 25.1  (15.7–40.1) | 31.3  (18.1–54.2) | 30.7  (17.5–53.9) | 343  (126–933) | 33.3*  (17.4–63.9) | 28.3  (17.1–46.9) | 444  (195–1013) | 40.7  (18.7–88.7) | 452  (223–915) |
| **Day 56** | 277  131–585) | 225  (104–485) | 567  (252–1272) | 247  (97.8–624) | 214  (92.2–497) | 533  (277–1028) | 347  (177–682) | 633  (315–1274) | 366  (198–677) | 770  (303–1956) | 264  (147–474) | 620  (393–977) | 280  (114–685) | 411  (240–704) |
| **Day 208** | 124  (63.8–242) | 96.2  (50.5–183) | 203  (98.8–417) | 126  (61.5–257) | 76.2  (37.4–155) | 173  (101–296) | 160  85.0–302) | 225  (125–403) | 169  (88.5–323) | 268  (123–584) | 115  (62.3–212) | 300  (190–475) | 118  (59.3–237) | 200*  (110–361) |
| **Day 393** | 74.1  (34.0–162) | 47.8  (25.0–91.1) | 101  (42.5–239) | 67.8  (32.2–143) | 42.3  (20.6–86.6) | 105  (58.2–190) | 106  (51.9–215) | 144  (84.4–246) | 83.9  (39.6–178) | 149  (66.2–334) | 55.0  (27.8–109) | 174  (96.2–316) | 80.0  (32.7–196) | 84.0  (45.2–156) |

| **Supplemental Table 5. Geometric HBGA BT_50_ (95% CI) against GII.4c** at the indicated days in each study group in the 18-49 and 50-64 year-old cohorts (Per protocol set) | | | | | | | | | | | | | | |
| --- | --- | --- | --- | --- | --- | --- | --- | --- | --- | --- | --- | --- | --- | --- |
| **Group** | **1** | **2** | **3** | **4** | **5** | **6** | **7** | **8** | | **9** | **10** | | **11** | |
|  |  |  |  |  |  |  |  | **8A** | **8B** |  | **10A** | **10B** | **11A** | **11B** |
| **HBGA BT_50_ vs. GII.4c in 18-49 year-olds** | | | | | | | | | | | | | | |
| **N** | 15 | 13 | 14 | 14 | 15 | 15 | 15 | 16 | 14 | 15 | 15 | 15 | 12 | 15 |
| **Day 1** | 79.6  (31.0–204) | 123  (56.4–270) | 187  (90.3–386) | 93.2  (40.9–212) | 125  (69.5–226) | 123  (57.5–261) | 70.9  (29.0–174) | 84.6  (43.2–166) | 65.7  (27.7–156) | 119  (50.1–283) | 135  (67.0–273) | 95.5  (48.8–187) | 96.7  (42.7–219) | 168  (84.5–333) |
| **Day 28** | 71.4  (28.4–180) | 104  (43.8–244) | 191  (90.6–401) | 93.9  (40.7–217) | 124  (66.6–232) | 124  (56.8–270) | 65.0  (28.6–147) | 89.0  (45.7–173) | 733  (396–1357) | 106  (43.9–256) | 137  70.1–266) | 940  (639–1383) | 90.9  (37.7–219) | 1134  (732–1760) |
| **Day 56** | 401  (191–841) | 759  (423–1363) | 655  (449–956) | 448  (291–691) | 1123  (656–1922) | 802  (577–1114) | 202  (80.0–510) | 635  (301–1340) | 546  (292–1023) | 433  (176–1065) | 1204  (860–1684) | 716  (523–982) | 812  (472–1397) | 827  (496–1378) |
| **Day 208** | 211  (111–400) | 323  (168–619) | 373  (262–532) | 254  (147–440) | 386  (214–694) | 297*  (176–503) | 137  (63.0–297) | 270  (136–537) | 353  (190–656) | 241  (113–510) | 438  (315–611) | 363  (246–536) | 306  (148–635) | 384*  (211–698) |
| **Day 393** | 140  (61.2–319) | 247*  (110–555) | 209  (120–364) | 141  (66.2–302) | 208  (121–355) | 177**  (91.2–345) | 96.6  (41.8–223) | 147  (69.1–312) | 164  (72.6–371) | 125  (57.5–272) | 264  (188–370) | 186  (108–319) | 191  (88.3–414) | 289*  (158–528) |
| **HBGA BT_50_ vs. GII.4c in 50-64 year-olds** | | | | | | | | | | | | | | |
| **N** | 14 | 16 | 15 | 16 | 15 | 16 | 14 | 16 | 14 | 14 | 15 | 14 | 12 | 14 |
| **Day 1** | 54.7  (24.1–124) | 52.4  (29.5–93.4) | 84.2  (37.0–192) | 81.1  (37.6–175) | 29.6  (15.0–58.3) | 77.3  (36.2–165) | 91.8  (41.9–201) | 95.3  (38.1–238) | 47.9  (27.3–84.0) | 76.5  (39.8–147) | 77.3  (41.0–146) | 88.3  (35.5–219) | 109  (56.3–211) | 95.0  (43.7–206) |
| **Day 28** | 67.2  (30.8–147) | 54.2  (29.6–99.1) | 87.6*  (38.3–200) | 76.5  (39.6–148) | 36.9  (18.4–73.9) | 73.3  (32.9–164) | 85.8  (39.2–188) | 108  (37.7–310) | 417  (178–976) | 91.8*  (49.5–170) | 111  (57.9–215) | 493  (214–1136) | 117  (59.1–232) | 687  (368–1283) |
| **Day 56** | 283  (152–525) | 633  (291–1375) | 281  (169–467) | 219  (109–441) | 407  (215–768) | 506  (326–784) | 177  (80.1–392) | 1118  (713–1753) | 333  (151–738) | 292  (164–519) | 619  (278–1374) | 490  (219–1095) | 740  (396–1384) | 442  (235–831) |
| **Day 208** | 165  (85.3–320) | 191  (98.9–368) | 152  (76.3–303) | 157  (86.7–285) | 137  (66.5–282) | 231  (132–403) | 149  (66.4–334) | 445  (298–666) | 218  (144–330) | 166  (86.0–320) | 265  (138–509) | 339  (187–613) | 349  (200–608) | 281*  (163–484) |
| **Day 393** | 103*  (45.4–233) | 130  (61.1–278) | 73.0  (32.1–166) | 126  (61.4–258) | 52.8  (23.9–117) | 107  (49.0–235) | 125*  (52.2–298) | 228  (124–416) | 97.0  (51.5–183) | 119  (54.2–260) | 122  (68.6–219) | 157  (63.0–391) | 234  (123–442) | 129*  (58.9–282) |

| **Supplemental Table 6.** | | Rates of solicited local reactions and systemic adverse events within 7 days of injection. | | | |
| --- | --- | --- | --- | --- | --- |
|  | **Day 1** | | | **Day 28** | |
| **Symptom** | **NoV*** | | **Havrix^®^** | **NoV (2^nd^ dose)*** | **NoV** |
|  |  |  |  |  |  |
| **N** | 88 | | 332 | 88 | 332 |
| **Pain** | 31  (36%) | | 146  (44.0%) | 32  (36%) | 161  (48.5%) |
| *(Severe)* | - | | *1 (0.3%)* | - | *2 (0.6%)* |
| **Erythema** | 0 | | 0 | 0 | 3  (0.9%) |
| **Induration** | 0 | | 0 | 0 | 6  (1.8%) |
| **Swelling** | 0 | | 1  (0.3%) | 0 | 5  (1.5%) |
| *(Severe)* | - | | *-* | - | *1 (0.3%)* |
| **Fever ^#^** | 0 | | 2  (0.6%) | 0 | 1  (0.3%) |
| *(Severe)* **^#^** | *-* | | *-* | *-* | *-* |
| **Headache** | 20  (22.7%) | | 60  (18.1%) | 20  (22.7%) | 54  (16.3%) |
| *(Severe)* | *3 (3.4%)* | | *1 (0.3%)* |  | *4 (1.2%)* |
| **Fatigue** | 19  (21.6%) | | 59  (17.8%) | 10  (11.4%) | 48  (14.5%) |
| *(Severe)* | *1 (1.1%)* | | *-* | - | *2 (0.6%)* |
| **Myalgia** | 10  (11.4%) | | 37  (11.1%) | 4  (4.5%) | 24  (7.2%) |
| *(Severe)* | - | | - | - | *1 (0.3%)* |
| **Arthralgia** | 3  (3.4%) | | 11  (3.3%) | 2  (2.3%) | 7  (2.1%) |
| *(Severe)* | *1 (1.1%)* | | *-* | - | - |
| **Vomiting** | 0 | | 4  (1.2%) | 0 | 2  (0.6%) |
| **Diarrhea** | 9  (10.2%) | | 38  (11.4%) | 5  (5.7%) | 37  (11.1%) |
| * Groups 8B, 10B and 11B **^#^** Fever = body temperature ≥ 38°C, severe ≥ 40°C | | | | | |
